# A curated list of genes that affect the plant ionome

**DOI:** 10.1002/pld3.272

**Published:** 2020-10-21

**Authors:** Lauren Whitt, Felipe Klein Ricachenevsky, Greg Ziegler Ziegler, Stephan Clemens, Elsbeth Walker, Frans J. M. Maathuis, Philip Kear, Ivan Baxter

**Affiliations:** ^1^ Donald Danforth Plant Science Center Saint Louis MO USA; ^2^ Departamento de Botânica Programa de Pós‐Graduação em Biologia Celular e Molecular Universidade Federal do Rio Grande do Sul Porto Alegre Brazil; ^3^ University of Bayreuth Bayreuth Germany; ^4^ University of Massachusetts Amherst Amherst MA USA; ^5^ University of York York United Kingdom; ^6^ Cornell University Ithaca NY USA

**Keywords:** curated, ionomics, mineral nutrition

## Abstract

Understanding the mechanisms underlying plants’ adaptation to their environment will require knowledge of the genes and alleles underlying elemental composition. Modern genetics is capable of quickly, and cheaply indicating which regions of DNA are associated with particular phenotypes in question, but most genes remain poorly annotated, hindering the identification of candidate genes. To help identify candidate genes underlying elemental accumulations, we have created the known ionome gene (KIG) list: a curated collection of genes experimentally shown to change uptake, accumulation, and distribution of elements. We have also created an automated computational pipeline to generate lists of KIG orthologs in other plant species using the PhytoMine database. The current version of KIG consists of 176 known genes covering 5 species, 23 elements, and their 1588 orthologs in 10 species. Analysis of the known genes demonstrated that most were identified in the model plant *Arabidopsis thaliana,* and that transporter coding genes and genes altering the accumulation of iron and zinc are overrepresented in the current list.

## 1 INTRODUCTION

Understanding the complex relationships that determine plant adaptation will require detailed knowledge of the action of individual genes, the environment, and their interactions. One of the fundamental processes that plants must accomplish is to manage the uptake, distribution, and storage of elements from the environment. Many different physiological, chemical, biochemical, and cell biology processes are involved in moving elements, implicating thousands of genes in every plant species. Modern genetic techniques have made it easy and inexpensive to identify hundreds to thousands of loci for traits, such as the elemental composition (or ionome) of plant tissues. However, moving from loci to genes is still difficult as the number of possible candidates is often extremely large and the ability of researchers to identify a candidate gene from its functional annotations is limited by our current knowledge and inherent biases about what is worth studying (Stoeger et al., 2018; Baxter, 2020).

The most obvious candidates for genes affecting the ionome in a species are orthologs of genes that have been shown to affect elemental accumulation in another species. Indeed, there are multiple examples of orthologs affecting elemental accumulation in distantly related species, such as *Arabidopsis thaliana* and rice (*Oryza sativa*), including Na^+^ transporters from the HKT family (Ren et al., 2005; Baxter et al., 2010); the heavy metal transporters AtHMA3 and OsHMA3 (Chao et al., 2012; Yan et al., 2016); E3 ubiquitin ligase BRUTUS and OsHRZs that regulate the degradation of iron uptake factors (Selote et al., 2015; Hindt et al., 2017; Kobayashi et al., 2013) and the K^+^ channel AKT1 (Ahmad et al., 2016; Lagarde et al., 1996). To our knowledge, no comprehensive list of genes known to affect elemental accumulation in plants exists. To ameliorate this deficiency, we sought to create a curated list of genes based on peer‐reviewed literature along with a pipeline to identify orthologs of the genes in any plant species and a method for continuously updating the list. Here we present version 1.0 of the known ionome gene (KIG) list.

## 2 MATERIALS AND METHODS

The list includes all functionally characterized genes from the literature that are linked to changes in the ionome. Criteria for inclusion in the primary KIG list were as follows:

1. The function or levels of the gene are unambiguously altered (i.e., a confirmed knockout, knockdown or overexpressor). For double mutants, both genes are listed.
2. The levels of at least one element are significantly altered in plant tissue.
3. Publication in the form of a peer‐reviewed manuscript.

Note that our definition excludes genes that are linked to metal tolerance or sensitivity but do not alter the ionome, or genes where the levels of the transcript are correlated with elemental accumulation. In order to identify the KIG genes, we created a Google survey that was distributed to members of the Ionomicshub research coordination network (NSF DBI‐0953433), as well as advertising on Twitter and in oral presentations by the authors. We asked submitters to provide the species, gene name (or names where alleles of two genes were required for a phenotype), gene ID(s), tissue(s), element(s) altered, and a DOI link for the primary literature support. Subsequently, authors FKR and LW did an extensive literature search.

### 2.1 Creating the inferred orthologs list

The known ionome gene list contains known genes from the primary list and their orthologous genes inferred by InParanoid (v4.1) pairwise species comparisons (Remm et al., 2001). The InParanoid files were downloaded from Phytozome for each organism‐to‐organism combination of species in the primary list, plus *Glycine max*, *Sorghum bicolor*, *Setaria italica*, *Setaria viridis,* and *Populus trichocarpa*. Orthologs of the primary genes were labeled as “inferred” genes. If a primary gene was also found as an ortholog to a primary gene in another species, the status was changed to “Primary/Inferred” in both species. It is important to note that only primary genes can infer genes; inferred genes cannot infer other genes. The pipeline for transforming the primary list into the known ionomics gene list can be found at https://github.com/baxterlab/KIG.

### 2.2 Gene Enrichment analysis

Overrepresentation analysis (released July 11, 2019) was performed on the primary and inferred genes in *A. thaliana* using the GO Consortium's web‐based GO Enrichment Analysis tool powered by the PANTHER (v14) classification system tool (Ashburner et al., 2000; Mi et al., 2017; The Gene Ontology Consortium, 2017). We restricted overrepresentation analysis to *A. thaliana* because of its dominance in the KIG list and our lack of confidence in the functional annotation of the other species on the list. An analysis performed by Wimalanathan et al. (2018) found that maize gene annotations in databases like Gramene and Phytozome lacked GO annotations outside of automatically assigned, electronic annotations (IEA). IEA annotations are not curated and have the least amount of support out of all the evidence codes (Harris et al., 2004). *A. thaliana* annotations come from a variety of evidence types, showing a higher degree of curation compared to maize (Wimalanathan et al., 2018). The whole‐genome *Arabidopsis thaliana* gene list from the PANTHER database was used as the reference list.

We tested both the PANTHER GO‐slim and the GO complete datasets for biological processes, molecular function, and cellular component. GO‐Slim datasets contain a selected subset of terms that give a broad summary of the gene list, whereas the complete dataset contains all the terms returned for a more detailed analysis. The enriched terms (fold enrichment >1 and with a false discovery rate <0.05) from the complete dataset were sorted into five specific categories relating to the ionome based annotation terms:

1. Ion homeostasis ‐ terms include homeostasis, stress, detoxification, regulation of an ion
2. Ion transport ‐ terms specifically state transport, export, import or localization of ion(s). Does not include hydrogen ion transport
3. Metal ion chelation ‐ terms relating to phytochelatins, other chemical reactions or pathways of metal chelator synthesis
4. Response to ions—vaguely states response to ions, but does not have any parent annotation terms that offer any more clarification (ie. stress response). Broadly this is referring to any change in the state or activity of cell secretion, expression, movement, or enzyme production (Carbon et al., 2009)
5. Other transport—annotation stating the transfer of anything that is not an ion (glucose, peptides, etc.)

Genes may belong to more than one category, but if they belong to a parent and child term in the same category, they are only counted once.

## 3 RESULTS

**FIGURE 1:**
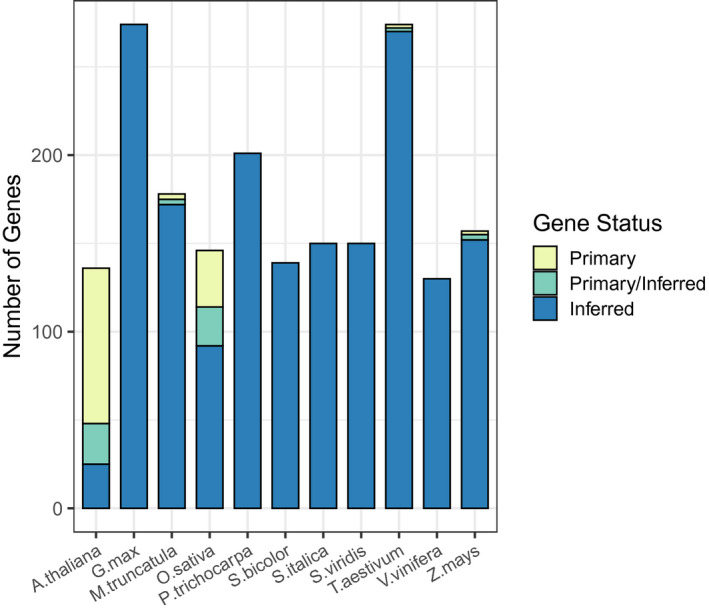
Number of genes for each species that are primary, inferred from other primary genes in other species, or both

The current primary list (v1.0) consists of 176 genes from *A. thaliana*, *O. sativa*, *Medicago truncatula*, *Triticum aestivum,* and *Zea mays* with the majority coming from *A. thaliana* and *O. sativa* (Table 1, Figure 1).

**TABLE 1:** Primary known ionome genes

Species	GeneID	GeneName	Elements	Tissue	Citation(s)
*A. thaliana*	AT1G01340	CNGC10	K, Ca, Mg	Roots, shoots	Guo et al. (2010)
*A. thaliana*	AT1G01580	FRO2	Fe	Root	Robinson et al. (1999)
*A. thaliana*	AT1G07600	MT1A	Cd, Zn, As	Shoots	Zimeri et al. (2005)
*A. thaliana*	AT1G08490	CPNIFS	Se, S	Roots, shoots	Van Hoewyk et al. (2005)
*A. thaliana*	AT1G12640	LPCAT1	P	Leaf	Kisko et al. (2018)
*A. thaliana*	AT1G14040	PHO1;H3	P	Shoots	Khan et al. (2014)
*A. thaliana*	AT1G14870	PCR2	Zn	Shoots	Song et al. (2010)
*A. thaliana*	AT1G18910	BTSL2	Fe, Mn, Zn	Leaf	Hindt et al. (2017)
*A. thaliana*	AT1G20110	FYVE1	Fe, Zn, Co, Mn	Root	Barberon et al. (2014)
*A. thaliana*	AT1G30270	CIPK23	K	Shoots	Xu et al. (2006)
*A. thaliana*	AT1G30400	ABCC1	Cd	Shoots	Park et al. (2012)
*A. thaliana*	AT1G30450	CCC	Ca, K, Na,S	seeds	McDowell et al. (2013)
*A. thaliana*	AT1G31885	NIP3;1	As	Shoots	Xu et al. (2015)
*A. thaliana*	AT1G32450	AtNRT1.5/ AtNPF7.3	K, NO3‐	Shoots, Roots	Li et al. (2017)
*A. thaliana*	AT1G36370	AtMSA1	S, Se	Shoots	Huang, et al. (2016)
*A. thaliana*	AT1G56160	myb72	Fe, Cd, Zn, Co, Mo	Leaf	Palmer et al. (2013)
*A. thaliana*	AT1G56430	NAS4	Fe, Cd, Co, Mo	Leaf	Palmer et al. (2013)
*A. thaliana*	AT1G59870	PEN3	Cd	Shoots, roots	Kim et al. (2007)
*A. thaliana*	AT1G60960	AtIRT3	Fe	Roots	Lin et al. (2009)
*A. thaliana*	AT1G62180	AtAPR2	S, Se	Shoots	Loudet et al. (2007); Chao, et al. (2014)
*A. thaliana*	AT1G63440	AtHMA5	Cu	Shoots	Andrés‐Colás et al. (2006)
*A. thaliana*	AT1G66240	AtAX1	Cu	Shoots	Shin et al. (2012)
*A. thaliana*	AT1G68320	MYB62	P	Roots, shoots	Devaiah et al. (2009)
*A. thaliana*	AT1G71200	AtCITF1	Cu	Shoots, Anthers	Yan et al. (2017)
*A. thaliana*	AT1G74770	BTSL1	Fe, Mn, Zn	Leaf	Hindt et al. (2017)
*A. thaliana*	AT1G76430	PHT1;9	P, As	Roots, shoots	Remy et al. (2012)
*A. thaliana*	AT1G80760	NIP6;1	B	Leaves,shoots	Tanaka et al. (2008)
*A. thaliana*	AT1G80830	AtNRAMP1	Mn	Shoots, roots	Cailliatte et al. (2010)
*A. thaliana*	AT2G01770	VIT1	Fe	Seed	Kim et al. (2006)
*A. thaliana*	AT2G01980	SOS1/NHX7	Na	Shoots	Shi et al. (2003)
*A. thaliana*	AT2G13540	ABH1	S	seeds	McDowell et al. (2013)
*A. thaliana*	AT2G16770	AtbZIP23	Zn	Shoots, roots	Assunção et al. (2010)
*A. thaliana*	AT2G19110	AtHMA4	Zn	Shoots, seeds	Hussain et al. (2004); Olsen et al. (2016)
*A. thaliana*	AT2G21045	AtHAC1	As	Shoots	Chao, et al. (2014)
*A. thaliana*	AT2G23150	AtNRAMP3	Fe, Mn, Zn	Shoots	Lanquar et al. (2010)
*A. thaliana*	AT2G23240	AtMT4b	Cu, Zn	Seeds	Ren et al. (2012)
*A. thaliana*	AT2G25680	MOT1	Mo	Leaf	Baxter, Muthukumar, et al., 2008; Baxter, Vitek, et al., 2008
*A. thaliana*	AT2G28160	FRU	Fe	Shoots	Yuan et al. (2008)
*A. thaliana*	AT2G28670	ESB1	Ca, Mn, Zn, Na, S, K, As, Se, Mo	Leaf	Baxter et al. (2009)
*A. thaliana*	AT2G32830	PHT1;5	P	Roots	Nagarajan et al. (2011)
*A. thaliana*	AT2G33770	PHO2	P	Roots, shoots	Liu et al. (2012)
*A. thaliana*	AT2G37430	ZAT11	Ni	Shoots	Liu et al. (2014)
*A. thaliana*	AT2G38460	FPN1	Co	Leaf	Morrissey et al. (2009)
*A. thaliana*	AT2G38940	PHT1;4	P	Roots, shoots	Shin et al. (2004)
*A. thaliana*	AT2G39450	AtMTP11	Mn	Shoots, roots	Peiter et al. (2007)
*A. thaliana*	AT2G42000	AtMT4a	Cu, Zn	Seeds	Ren et al. (2012)
*A. thaliana*	AT2G46430	CNGC3	K	Leaf	Gobert et al. (2006)
*A. thaliana*	AT2G46800	AtMTP1	Zn	Shoots	Desbrosses‐Fonrouge et al. (2005)
*A. thaliana*	AT2G47160	BOR1	B	Shoots	Miwa et al. (2006)
*A. thaliana*	AT3G01310	VIH2	P	Shoots	Zhu et al. (2019)
*A. thaliana*	AT3G06060	TSC10a	Na, K, Rb, Mg, Ca, Fe, Mo	Leaf	Chao et al. (2011)
*A. thaliana*	AT3G06100	NIP7	As	NA	Lindsay and Maathuis (2016; Isayenkov and Maathuis (2008)
*A. thaliana*	AT3G08040	FRD3/MAN1	Mn	Leaf	Delhaize (1996)
*A. thaliana*	AT3G12750	AtZIP1	Mn	Roots	Milner et al. (2013)
*A. thaliana*	AT3G12820	myb10	Fe, Cd, Zn, Co, Mo	Leaf	Palmer et al. (2013)
*A. thaliana*	AT3G13320	CAX2	Mn, Fe, K, P	Seed	Connorton et al. (2012)
*A. thaliana*	AT3G13405	mir169a	N	Root	Zhao et al. (2011)
*A. thaliana*	AT3G14280		S	seeds	McDowell et al. (2013)
*A. thaliana*	AT3G15380	AtCTL1	Na, Fe, Zn, Mn, Mo	Shoots, Roots	Gao et al. (2017)
*A. thaliana*	AT3G18290	BTS	Fe, Zn, Mn	Leaf	Hindt et al. (2017)
*A. thaliana*	AT3G22890	AtATPS1	S	Shoos	Koprivova et al. (2013)
*A. thaliana*	AT3G23210	bHLH34	Fe	Root, shoot	Li et al. (2016)
*A. thaliana*	AT3G23430	PHO1	P	Shoots	Khan et al. (2014)
*A. thaliana*	AT3G43790	ZIFL2	Cs	Leaf	Remy et al. (2015)
*A. thaliana*	AT3G47640	PYE	Fe, Zn, Mn, Co	Root	Long et al. (2010)
*A. thaliana*	AT3G47950	AHA4	Na	Root	Vitart et al. (2001)
*A. thaliana*	AT3G51860	CAX3	P, K	Seed	Connorton et al. (2012)
*A. thaliana*	AT3G51895	SULTR3;1	S	Leaf	Cao et al. (2013)
*A. thaliana*	AT3G56970	bHLH38	Fe	Shoots	Yuan et al. (2008)
*A. thaliana*	AT3G56980	bHLH39	Fe	Shoots	Yuan et al. (2008)
*A. thaliana*	AT3G58060	AtMTP8	Mn	Shoots, seeds	Eroglu et al. (2016, Eroglu et al. (2017)
*A. thaliana*	AT3G58810	AtMTP3	Zn	Shoots	Arrivault et al. (2006)
*A. thaliana*	AT3G58970	MGT6	Mg	Roots, shoots	Mao et al. (2014)
*A. thaliana*	AT3G62270	BOR2	B	Shoots	Miwa et al. (2013)
*A. thaliana*	AT4G02780	GA1	Fe	Root	Wild et al. (2016)
*A. thaliana*	AT4G10310	AtHKT1;1	Na	Leaf	Baxter et al. (2010)
*A. thaliana*	AT4G10380	NIP5;1	B	Roots, shoots	Takano et al. (2006)
*A. thaliana*	AT4G13420	HAK5	Rb, Cs	Roots	Rubio et al. (2008; Qi et al. (2008)
*A. thaliana*	AT4G14410	bHLH104	Fe	Root, shoot	Li et al. (2016)
*A. thaliana*	AT4G16370	OPT3	Fe, Cd	Leaf	Zhai et al. (2014)
*A. thaliana*	AT4G19690	IRT1	Fe, Mn, Co, Cd, Zn	Root	Eide et al. (1996)
*A. thaliana*	AT4G23100	GSH1	Cd, As	Shoots	Guo et al. (2008)
*A. thaliana*	AT4G24120	YSL1	Fe, Zn, Cu	NA	Waters et al. (2006)
*A. thaliana*	AT4G28610	AtPHR1	P	Shoots	Nilsson et al. (2007)
*A. thaliana*	AT4G30110	AtHMA2	Zn	Shoots, seeds	Hussain et al. (2004; Olsen et al. (2016)
*A. thaliana*	AT4G30120	AtHMA3	Cd, Zn	Leaf	Chao et al. (2012; Pita‐Barbosa et al. (2019)
*A. thaliana*	AT4G33000	CBL10	K	Shoots	Ren et al. (2013)
*A. thaliana*	AT4G35040	AtbZIP19	Zn	Shoots, roots	Assunção et al. (2010)
*A. thaliana*	AT4G37270	HMA1	Zn	Shoots	Kim et al. (2009)
*A. thaliana*	AT5G02600	NaKR1	Na, K, Rb	Leaf	Tian et al. (2010)
*A. thaliana*	AT5G03455	ACR2	As, P	Roots, shoots	Dhankher et al. (2006)
*A. thaliana*	AT5G03570	FPN2	Co, Ni	Leaf	Morrissey et al. (2009); Schaaf et al. (2006)
*A. thaliana*	AT5G09690	MGT7	Mg	Shoots	Kamiya et al. (2012)
*A. thaliana*	AT5G13740	ZIF1	Zn, Fe	Shoots	Haydon et al. (2012)
*A. thaliana*	AT5G15070	VIH1	P	Shoots	Zhu et al. (2019)
*A. thaliana*	AT5G15410	CNGC2/DND1	Ca, Mg	seeds	McDowell et al. (2013)
*A. thaliana*	AT5G17290	APG5	Fe, Mn, Zn	Leaf, shoots, seeds	Pottier et al. (2019)
*A. thaliana*	AT5G18830	AtSPL7	Cu	Shoots, roots	Bernal et al. (2012)
*A. thaliana*	AT5G20650	COPT5	Cu	Shoots, roots, seeds	Klaumann et al. (2011)
*A. thaliana*	AT5G35410	SOS2	Na	Seeds	McDowell et al. (2013)
*A. thaliana*	AT5G42130	AtMfl1	Fe	Leaves, shoots	Tarantino et al. (2011)
*A. thaliana*	AT5G43350	PHT1;1	P, As	Shoots	Shin et al. (2004; Catarecha et al. (2007)
*A. thaliana*	AT5G44070	PCS1	Zn, Cd, As	Leaf	Kühnlenz et al. (2016; Guo et al. (2008)
*A. thaliana*	AT5G53130	CNGC1	Pb	Leaf	Sunkar et al. (2000)
*A. thaliana*	AT5G53550	YSL3	Fe, Zn, Cu	NA	Waters et al. (2006)
*A. thaliana*	AT5G54680	ILR3	Cd, Co, Fe, Mn, Zn	Leaf	Rampey et al. (2006)
*A. thaliana*	AT5G54810	AtTSB1	Cd	Roots,shoots	Sanjaya et al. (2008)
*A. thaliana*	AT5G57620	AtMYB36	Li, B, Na, Mg, K, Ca, Mn, Fe, Co, Ni, Cu, Zn, Rb, Sr, Mo, Cd	Shoots	Kamiya et al. (2015)
*A. thaliana*	AT5G59030	COPT1	Cu	Seed, Leaf	Sancenón et al. (2004)
*A. thaliana*	AT5G64930	CPR5	K	Leaf	Borghi et al. (2011)
*A. thaliana*	AT5G67330	AtNRAMP3	Fe, Mn, Zn	Shoots	Lanquar et al. (2010)
*M. truncatula*	Medtr1g010270	MtMOT1.2	Mo	Nodules	Gil‐Díez et al. (2018)
*M. truncatula*	Medtr3g088460	MtNramp1	Fe	Nodules	Tejada‐Jiménez et al. (2015)
*M. truncatula*	Medtr3g464210	MtMOT1.3	Mo	Nodules	Tejada‐Jiménez et al. (2017)
*M. truncatula*	Medtr4g019870	MtCOPT1	Cu	Nodules	Senovilla et al. (2018)
*M. truncatula*	Medtr4g064893	MtMTP2	Zn	Nodules	León‐Mediavilla et al. (2018)
*M. truncatula*	Medtr4g083570	MtZIP6	Zn	Nodules	Abreu et al. (2017)
*O. sativa*	LOC_Os01g03914	OsMTP9	Mn	Shoots	Ueno et al. (2015)
*O. sativa*	LOC_Os01g20160	OsHKT1;5	Na	Leaf, shoots	Kobayashi et al. (2017)
*O. sativa*	LOC_Os01g45990	AKT1	K	NA	Ahmad, et al. (2016)
*O. sativa*	LOC_Os01g64250	OsHORZ1	Fe	Shoots,seeds	Kobayashi et al. (2013)
*O. sativa*	LOC_Os01g64890	OsMGT1	Mg,Na	Roots, shoots	Chen, et al. (2017)
*O. sativa*	LOC_Os02g06290	OsHAC4	As	Seed	Xu et al. (2017)
*O. sativa*	LOC_Os02g10290	OsHMA4	Cu	Roots, shoots, seeds	Huang, et al. (2016)
*O. sativa*	LOC_Os02g13870	OsNIP1;1	As	Shoots	Sun et al. (2018)
*O. sativa*	LOC_Os02g43370	OsYSL2	Fe, Mn	Seeds	Ishimaru et al. (2010)
*O. sativa*	LOC_Os02g43410	OsYSL15	Fe	Roots, shoots, seeds	Lee et al. (2009)
*O. sativa*	LOC_Os02g51110	LSI1	Se	Roots, shoots	Zhao et al. (2010)
*O. sativa*	LOC_Os02g53490	OsMTP8.2	Mn	Shoots, roots	Takemoto et al. (2017)
*O. sativa*	LOC_Os02g56510	OsPHO1;2	P	Shoots	Secco et al. (2010)
*O. sativa*	LOC_Os03g05640	OsPT2	Se	Roots, shoots	Zhang et al. (2014)
*O. sativa*	LOC_Os03g09140	OsRab6a	Fe, Zn	Seeds, shoot, roots	Yang and Zhang (2016)
*O. sativa*	LOC_Os03g12530	OsMTP8.1	Mn	Shoots, roots	Chen et al. (2013)
*O. sativa*	LOC_Os03g18550	OsMIT	Fe	Shoots	Bashir et al. (2011)
*O. sativa*	LOC_Os03g19420	OsNAS2	Fe	Seeds	Lee et al. (2012)
*O. sativa*	LOC_Os03g21240	OsPHR2	P	Shoots	Zhou et al. (2008)
*O. sativa*	LOC_Os04g32920	OsHAK1	Cs	Shoots, seeds	Rai et al. (2017)
*O. sativa*	LOC_Os04g38940	OsVIT1	Fe,Zn	Shoots, seeds	Zhang et al. (2012)
*O. sativa*	LOC_Os04g45860	OsYSL9	Fe	Shoots, seeds	Senoura et al. (2017)
*O. sativa*	LOC_Os04g45900	OsYSL16	Cu	Roots, shoots, seeds	Zheng et al. (2012)
*O. sativa*	LOC_Os04g46940	OsHMA5	Cu	Roots,s hoots	Deng et al. (2013)
*O. sativa*	LOC_Os04g52310	OsZIP3	Zn	Shoots	Sasaki et al. (2015)
*O. sativa*	LOC_Os04g52900	OsABCC1	As	Seeds	Song et al. (2014)
*O. sativa*	LOC_Os04g56430	OsRMC	Fe,Mn,Cu	Root, shoot, seeds	Yang et al. (2013)
*O. sativa*	LOC_Os05g34290	OsPCS1*	As	Seeds	Hayashi et al. (2017)
*O. sativa*	LOC_Os05g39560	OsZIP5	Zn	Leaf	Lee et al. (2010)
*O. sativa*	LOC_Os05g47780	OsHRZ2	Fe	Shoots, seeds	Kobayashi et al. (2013)
*O. sativa*	LOC_Os05g48390	OsPHO2	P	Leaf	Wang et al. (2009)
*O. sativa*	LOC_Os06g01260	OsPCS2*	As, Cd	Seeds	Uraguchi et al. (2017)
*O. sativa*	LOC_Os06g05160	SPDT	P	Seed	Yamaji et al. (2017)
*O. sativa*	LOC_Os06g48720	OsHMA2	Zn	Shoots, roots	Takahashi et al. (2012)
*O. sativa*	LOC_Os06g48810	OsHKT2;1	Na	Roots, shoots	Horie et al. (2007)
*O. sativa*	LOC_Os07g01810	TPKb	K	Leaf, root	Ahmad et al. (2016)
*O. sativa*	LOC_Os07g09000	OsPHF1	P	Leaf, root	Chen et al. (2011)
*O. sativa*	LOC_Os07g12900	OsHMA3	Cd	Shoots, seeds	Tanaka et al. (2016)
*O. sativa*	LOC_Os07g15370	NRAMP5	Fe,Mn,Cd	Leaf	Sasaki et al. (2012)
*O. sativa*	LOC_Os08g01120	OsMOT1;1	Mo	Shoots, Seed	Huang et al. (2019)
*O. sativa*	LOC_Os08g04390	OsPRI1	Fe	Shoots, roots	Zhang et al. (2017)
*O. sativa*	LOC_Os08g05590	OsNIP3;2	As	Roots	Chen, Sun, et al. (2017a); Chen, Yamaji, et al. (2017b)
*O. sativa*	LOC_Os08g05600	OsNIP3;3	As	Shoots	Sun et al. (2018)
*O. sativa*	LOC_Os08g10480	OsATX1	Cu	Shoots, roots, seeds	Zhang, Cao, et al. (2018); Zhang, Chen, et al. (2018)
*O. sativa*	LOC_Os09g23300	OsVIT2	Fe, Zn	Shoots, seeds	Zhang et al. (2012)
*O. sativa*	LOC_Os12g03899	ZIFL12	Fe	Shoots	Che et al. (2019)
*O. sativa*	LOC_Os12g18410	OsMIR	Fe	Shoots, Roots, seeds	Ishimaru et al. (2009)
*O. sativa*	LOC_Os12g32400	OsbHLH133	Fe	Leaf, root, shoot	Wang, Sun, et al. (2013a); Wang, Ying, et al. (2013b)
*O. sativa*	LOC_Os12g37840	OsBOR1	B	Shoots	Nakagawa et al. (2007)
*O. sativa*	Os01g0689300	OsHRZ1	Fe	Shoots, seeds	Kobayashi et al. (2013)
*T. aestivum*	2Al‐TRIAE_CS42_ 2AL_TGACv1_095050_AĂ410	TaIPK1	Fe, Zn	Seed	Aggarwal et al. (2018)
*T. aestivum*	Traes_4AS_7220D33B3	Ta‐PHR1	P	Shoots	Wang, Sun, et al. (2013a); Wang, Ying, et al. (2013b)
*T. aestivum*	Traes_4BL_7091749BF	TaABCC13	Ca	Seed	Bhati et al. (2016)
*T. aestivum*	Traes_4DL_3F8034BFD	HKT2;1	Na	Roots	Laurie et al. (2002)
*Z. mays*	GRMZM2G047616	ZmHKT1	Na	Leaf	Zhang, Cao, et al. (2018); Zhang, Chen, et al. (2018)
*Z. mays*	GRMZM2G060952	YS1	Fe	Root	Von Wiren et al. (1994)
*Z. mays*	GRMZM2G063306	YS3	Fe	Leaf	Chan‐Rodriguez and Walker (2018)
*Z. mays*	GRMZM2G084779	ZmHAK5	K	Roots, shoots	Qin et al. (2019)
*Z. mays*	GRMZM2G176209	TLS1	B	Shoots, roots, anthers	Durbak et al. (2014)

Most primary genes have orthologs in other species. Less than 10% of primary genes in *A. thaliana*, 12% in *O. sativa,* and one of the four primary genes in wheat (*T. aestivum*) lack orthologs (Table 2). *G. max*, *P. trichocarpa*, *S. bicolor*, *S. italica*, and *S. viridis* currently contain only inferred genes (Table 2, Figure 1).

**TABLE 2:** Break down of primary/inferred genes in each species

Species	Total genes	Primary genes	Primary/inferred genes	Inferred genes	Primary & primary/inferred genes without orthologs
*A. thaliana*	136	65.44%	16.18%	18.38%	9.91%
*O. sativa*	141	20.57%	14.89%	64.54%	12.00%
*M. truncatula*	176	1.70%	1.70%	96.59%	0.00%
*T. aestivum*	267	0.75%	0.75%	98.50%	25.00%
*Z. mays*	152	1.32%	1.97%	96.71%	0.00%
*G. max*	268	0.00%	0.00%	100.00%	0.00%
*P. trichocarpa*	197	0.00%	0.00%	100.00%	0.00%
*S. bicolor*	135	0.00%	0.00%	100.00%	0.00%
*S. italica*	146	0.00%	0.00%	100.00%	0.00%
*S. viridis*	146	0.00%	0.00%	100.00%	0.00%

The YSL genes in *A. thaliana* and *O. sativa* are an example that provides evidence for the validity of the KIG list pipeline: AtYSL3, OsYSL9, and OsYSL16 were listed in their respective species as primary genes (Table 1) and after the ortholog search was annotated as primary/inferred genes, referencing each other (Table S1). AtYSL2 in *A. thaliana*, was not listed as primary gene, but was inferred through its rice orthologs OsYSL9 and OsYSL16. Additionally, AtYSL1 in *A. thaliana* is not a paralog of AtYSL3 or an ortholog of OsYSL9 and OsYSL16 according to PhytoMine's InParanoid results and is not listed as an ortholog to either of the *O. sativa* YSL genes in the KIG list. Other examples include AtVIT1 and OsVIT1/OsVIT2 (Kim et al., 2006; Zhang et al., 2012), and the vacuolar Mn transporters AtMTP8 and OsMTP8 (Eroglu et al., 2016; Chen et al., 2013). Thus, we can reliably generate inferred genes and create a species‐specific KIG list for any species in PhytoMine.

**FIGURE 2:**
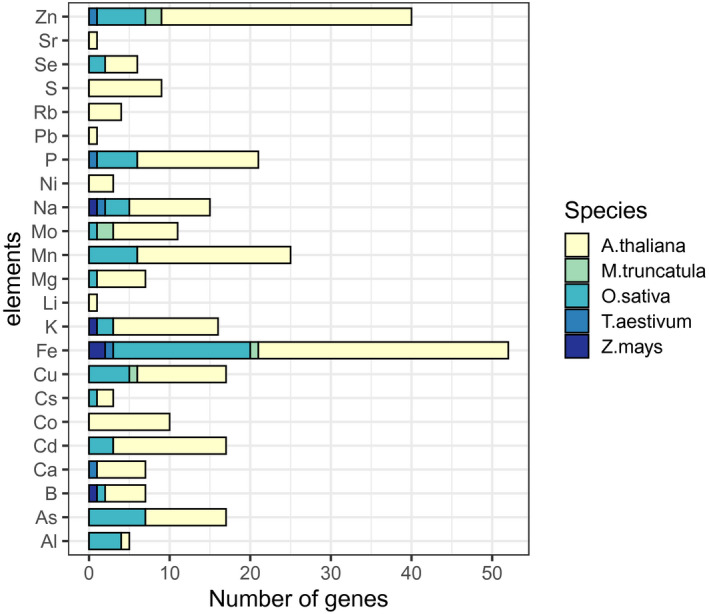
Number of primary genes from each species listing each element

The primary list covers 23 elements (Figure 2) according to the reported elements from authors in the primary list, which is more elements than predicted by the GO term annotations for those genes. Some GO annotations for these genes mention only a portion of elements listed by the literature on the primary list. This may be due to GO annotation evidence codes lacking curation or biological data (IEA, ND, NAS) (Wimalanathan et al., 2018), or it may be due to alterations in one element leading to alterations in other elements (Baxter, Muthukumar, et al., 2008; Baxter, Vitek, et al., 2008).

**FIGURE 3:**
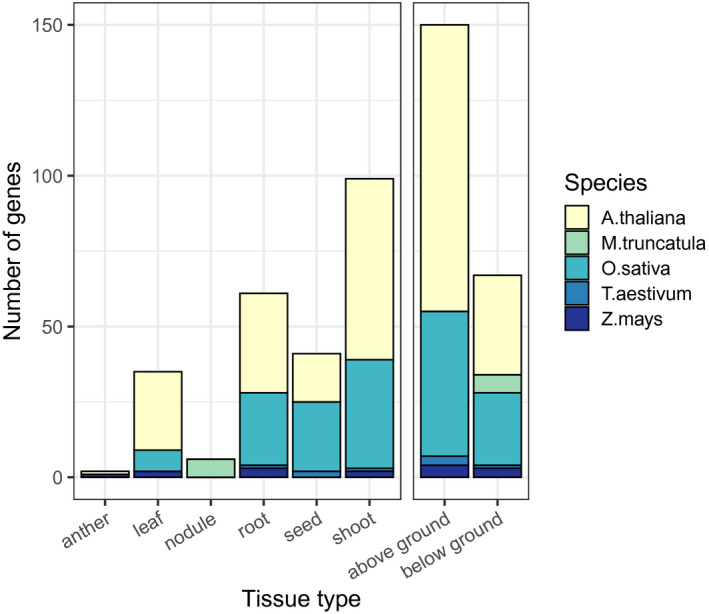
Number of primary genes each type of tissue contributes to the known ionome gene list. Above ground is a summary of anther, leaf, seed, and shoot, while below ground is a summary of root and nodule

*A. thaliana* is the only species to have a primary gene listing for each element. There is a bias toward manganese, zinc, and iron which have two, three, and four times more associated genes than the average 13 ± 12 genes of other elements. Iron is the only element to contain genes from all five species in the primary list. In addition to biases toward certain elements, our primary list is also skewed toward an overrepresentation of ionome genes in above‐ground tissue studies (Figure 3). This is likely due to the difficulties in studying the elemental content of below‐ground tissues. All *M. truncatula* genes come from studies of the nodule in this model legume species.

**FIGURE 4:**
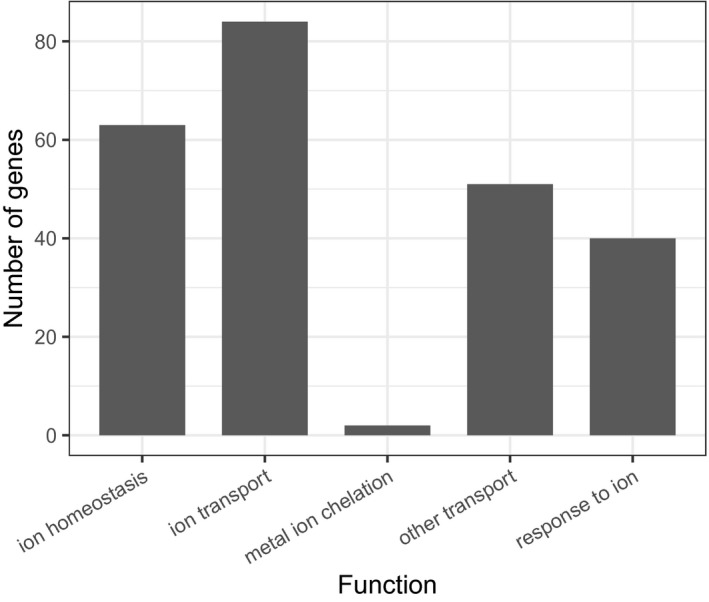
Known ionome genes relating to different terms from the GO complete biological process dataset. Ontology groups of GO Enrichment Analysis from PANTHER

Querying the manually curated PANTHER GO‐slim biological process database (PANTHER v14.1, released March 12, 2019) and the GO complete biological process database (GO Ontology database, released October 08, 2019) with the *A. thaliana* KIG genes returned significantly (FDR < 0.05) overrepresented annotation terms related to the transport, response, and homeostasis of iron, zinc, copper and manganese ions. Additionally, the GO complete results had terms for cadmium, nickel, cobalt, sulfur, arsenic, lead, selenium, boron, magnesium, phosphorus, sodium, potassium, and calcium; covering most of the elements in the KIG list (Figure 4). Even though some genes were annotated as associated in the “other transport” of glycoside, glucose, oligopeptides, or phloem transport, the citations that have added them into our primary list show that their mutant alleles altered elemental accumulation. AtABCC1 is annotated as encoding a glycoside transporter protein, but Park et al. (2012) found overexpression of AtABCC1 increased cadmium concentrations in shoot tissue. The YSL genes and OPT3 are annotated as genes encoding oligopeptide transporters, but more specifically they are encoding predicted phloem‐localized metal‐nicotianamine complex and iron/cadmium transporters, respectively (Waters et al., 2006; Zhai et al., 2014). Last, NRT1.5/NPF7.3 is also annotated as encoding an oligopeptide transporter, but Li et al., (2017) identified it as a xylem loading potassium ion antiporter.

The PANTHER GO‐slim molecular function annotation database found a significant overrepresentation for iron and potassium cation transmembrane transporter activity in the *A. thaliana* genes. The results using the GO complete molecular function database supported this and additionally included terms for arsenic, cadmium, zinc, boron, manganese, phosphate, sulfur, and magnesium ion transmembrane transporter activity. The GO complete molecular database also returned overrepresented terms for metal ion‐binding and cyclic nucleotide‐binding annotations. The cyclic nucleotide‐binding annotation genes were more specifically cyclic nucleotide ion gated channel genes (Gobert et al., 2006). The PANTHER GO‐slim cell component and GO complete cell component annotation database both returned significant overrepresentation for vacuoles and the plasma membrane, both known to be critical for elemental movement and storage (Barkla & Pantoja, 1996). The molecular function and cell component results are further evidence that our list is dominated by ion transporters.

To test the completeness of the KIG list, we searched PANTHER’s biological processes annotations for the number of *A. thaliana* genes encoding predicted elemental transporters. We found 618 *A. thaliana* genes predicted to encode elemental transport, and only 40 of these PANTHER genes are listed in the KIG list. We checked these results against ThaleMine (v1.10.4, updated on June 13, 2017) genes with the term “ion transport” in the gene name, description, or GO annotation and found only 358 genes, with 52 of these genes listed in the *A. thaliana* known ionome gene list. Interestingly, 219 of the genes from ThaleMine were not found in the 634 from PANTHER.

## 4 DISCUSSION

Here we have produced a curated list of genes known to alter the elemental composition of plant tissues. We envision several possible uses for this list:

1. Researchers can use the list to identify candidate genes in loci from QTL and GWAS experiments.
2. This list can serve as a gold standard for computational approaches.
3. The list can serve as a reading list for those interested in learning about elemental accumulation.

It is important to highlight that the inferred genes lists are not likely to be perfect predictors of the causal genes. Our use of InParanoid orthologs may exclude homologs that are likely candidates. Additionally, the reasons that some genes have been studied could be the result of human bias toward research topics (Baxter, 2020). The list is highly enriched for (a) transporters, (b) genes that affect elemental accumulation in above‐ground tissues, and (c) genes that affect the accumulation of Fe and Zn. Transporter genes became obvious candidates for studying plant nutrition when disruption allele collections were produced (McDowell et al., 2013). Above‐ground tissues are easier to study without contamination from the soil, and such studies are, therefore, more prevalent. Finally, while Fe and Zn are important biochemical cofactors, these elements are likely enriched in the KIG list due to their considerable interest in the community where the ionomics approach was developed. This is further illustrated in the PANTHER GO‐slim databases, where Fe was the only element to have its overrepresented response, homeostasis, and transport‐related GO terms show up in the PANTHER GO‐slim biological process and molecular function databases, which are selected subsets of terms meant to broadly summarize data. Overrepresented terms related to other KIG list elements are only found in the GO complete databases. Taken together, these factors warn against forming conclusions about the nature of all elemental accumulation genes based on this limited dataset.

Most entries on this list are derived from model organisms, suggesting that most of our knowledge about genes that affect elemental accumulation comes from these species. *A. thaliana* and *M. truncatula* account for 64% of the primary genes list, which is probably a lower bound for the influence of knowledge generated in model organisms. Several of the genes in crop plants were found due to being orthologs of genes in the model organisms (Ahmad, et al., 2016; Xu et al., 2017), and on closer inspection of the 50 papers identifying primary genes in rice, 38 cited a gene in Arabidopsis (not necessarily the direct ortholog) as a source for why the gene was investigated. The higher quality of the GO terms in Arabidopsis, when compared to other species, is another reflection of this disparity of knowledge and a significant hindrance when trying to clone genes in other organisms.

### 4.1 Call for more submissions

While we have done our best to ensure that the current list is useful and thorough, it is possible we are still missing genes. We ask readers who know of genes that we are missing to contribute by submitting them here: https://docs.google.com/forms/d/e/1FAIpQLSdmS_zeOlxTOLmq2wB45BuSQml1LMKtKnWSatmFRGR2Q1o0Ew/viewform?c=0&w=1 or email corresponding author. KIG lists v1.0 for each of the species can be viewed in Table S1, and future updates to the list can be found at https://docs.google.com/spreadsheets/d/1XI2l1vtVJiHrlXLeOS5yTQQnLYq7BOHpmjuC‐kUejUU/edit?usp=sharing.

## AUTHORS CONTRIBUTIONS

Contributed genes: IB, FKR, FM, SC, EW, PK. Analyzed data: LW, GZ. Wrote paper: LW, FKR, IB. Edited paper: FKR, FM, SC, EW, PK, GZ, LW, IB.

## Supporting information

Table S1
